# Human papillomavirus genotypes and HPV-16 variants distribution among Tunisian women with normal cytology and squamous intraepithelial lesions

**DOI:** 10.1186/s13027-016-0109-2

**Published:** 2016-12-01

**Authors:** R. Ghedira, W. Mahfoudh, S. Hadhri, S. Gabbouj, I. Bouanene, H. Khairi, A. Chaieb, R. Khelifa, N. Bouaouina, S. Remadi, A. A. Elmi, D. Bansal, A. A. Sultan, R. Faleh, A. Zakhama, L. Chouchane, E. Hassen

**Affiliations:** 1 Molecular Immuno-Oncology Laboratory, Monastir University, Monastir, Tunisia; 2 Faculty of Sciences, Carthage University, Bizerte, Tunisia; 3 National Office of Family and Population, Monastir, Tunisia; 4 Department of Epidemiology and preventive medicine, Faculty of Medicine, Monastir University, Monastir, Tunisia; 5 Department of Gynecology Obstetrics, Farhat Hached University Hospital, Sousse, Tunisia; 6 Unit of Viral and Molecular Tumor Diagnostics, Habib Thameur Hospital, Tunis, Tunisia; 7 Department of Cancerology Radiotherapy, Farhat Hached University Hospital, Sousse, Tunisia; 8 Laboratory of Anatomy and Pathologic cytology, Sousse, Tunisia; 9 Department of Microbiology and Immunology, Weill Cornell Medicine-Qatar, Cornell University, Doha, Qatar; 10 Department of Gynecology and Obstetrics, University Hospital of Monastir, Monastir, Tunisia; 11 Laboratory of Genetic Medicine and Immunology, Weill Cornell Medicine-Qatar, Cornell University, Doha, Qatar; 12 Higher Institute of Biotechnology of Monastir, Monastir University, Monastir, Tunisia

**Keywords:** Human Papillomavirus, Cervix, Prevalence, HPV-16 variants, Tunisian women

## Abstract

**Background:**

Little is known about the epidemiological characteristics of papillomavirus (HPV) infection among North African countries. Herein, we conducted a molecular epidemiological study to investigate prevalence of HPV type and HPV-16 variants among cervical-screened unvaccinated Tunisian women.

**Methods:**

Cross-sectional study was performed on 494 Tunisian women visiting Women’s Healthcare Centers. HPV-DNA detection was carried out on cervical samples using real-time polymerase chain reaction. HPV genotyping and HPV-16 variants were characterized by direct sequencing of *L1* viral capsid gene.

**Results:**

The overall HPV prevalence was 34% (95% CI: 30–38%) with significantly higher prevalence among women with squamous intraepithelial lesions (SIL) than those with no intraepithelial lesions (NIL) 84% (95% CI: 76–92%) and 24.5% (95% CI: 20–29%) respectively. The distribution of HPV prevalence according to women’s age shows a U-shaped curve and the highest HPV prevalence rates were observed among the youngest (≤25 years; 51.2%, 95% CI: 37–67%) and the oldest women (>55 years; 41.7%, 95% The HPV-16 prevalence was 32.8% (95% CI: 22–45%) among women with SIL and 9.2% (95% CI: 6–12%) among women with NIL. Whereas, the HPV-18 prevalence was 1.3% (95% CI: 0–5%) among women with SIL and 0.3% (95% CI: 0–1%) among women with NIL. Among HPV-16 positive women, European lineage (E) was identified as the predominant HPV-16 variant (85.7%, 95% CI: 76–95%). The frequency of E variant was lower among SIL than among NIL women (81%, 95% CI: 64–99%, and 88%, 95% CI: 77–100%, respectively). Conversely, the African-2 variant frequency was higher among SIL than among NIL women (18%, 95% CI: 1–36% and 6%, 95% CI: 2–14%, respectively). In multivariate analysis, young age was the only risk factor that is independently associated with HPV infection. Moreover, HPV infection and menopause were both found to be independently associated with SIL and HSIL.

**Conclusion:**

HPV DNA testing should be proposed to young and menopausal Tunisian women. Considering HPV prevalence, only 13% of the Tunisian women could be protected by the bivalent HPV vaccine. These results may be helpful for designing an adapted HPV testing and vaccination program in Tunisia.

## Background

According to the International Agency for Research on Cancer (IARC) 528,000 new cases of cervical cancer were estimated worldwide in 2012 [1]. In Tunisia, where Human Papillomavirus (HPV) screening and vaccination against HPV infection are not common, cervical cancer represents a major public health problem, with an estimated 265 new diagnosed cancer cases and 103 deaths in 2012 [1]. Several clinical and molecular epidemiological studies demonstrated that both HPV infection and persistent infection with high-risk HPV (HR-HPV) are the leading cause for the progression to cervical lesions [2, 3]. Papillomavirus are small, non-enveloped viruses that belong to the *Papillomaviridae* family with approximately 8 Kbp circular double-stranded DNA. The genome encodes for 6 early proteins responsible for virus replication and 2 late proteins L1 and L2, which are the viral structural proteins [4, 5]. According to De Villiers et al, determination of HPV types, subtypes and variants are based on L1 gene sequence similarities [6]. To date, more than 200 different HPV genotypes have been identified [7], of which 30 to 40 infect the genital tract through sexual contact and are classified on the basis of their oncogenic potential as HR-HPV and low-risk HPV (LR-HPV) [8, 9]. The most frequent HR types, HPV-16 and -18, are associated with approximately 70% of cervical cancer cases worldwide [10]. HPV-16 variants have been well characterized and can be divided into 4 phylogenetic variants: European (E), Asian-American (AA), African-1 (Af-1) and African-2 (Af-2) [11]. Several reports have described that HPV-16 variants differ in their pathogenicity and can affect the persistence of HPV infection and its progression from cervical precancerous lesion to cervical cancer. For instance, HPV-16 non-European variants were more pathogenic in high-grade cervical lesions than European variants [12, 13].

The age specific HPV prevalence has varied widely across different population and showed a bimodal distribution with two peaks of HPV positivity in younger and older women, respectively [14–16]. In addition, a meta-analysis including 78 studies reported that, in Africa, Europe and America, a clear second peak of HPV infection was observed in women older than 45 years [17]. As HPV infection is sexually transmitted, the HPV-associated cervical lesions are associated with several sexual habits and a particular lifestyle, such as younger age at first sexual intercourse, multi-sexual partners, smoking and oral contraceptive use [18, 19].

So far, few molecular epidemiological studies on HPV infections have been conducted in North-African countries, which is a region located at a crossroad of Southern Europe, Sub-Saharan Africa and the broader Middle East [20–22]. This location allows meeting, crossing and interaction of the different Mediterranean civilizations that had lived in North Africa along several centuries (Berber, Arabo-Islamic, Ottoman and European). Therefore, the current North-African social and cultural habits are a mixture of influences from these very different cultures that may give a peculiar profile of risk factors to HPV infection and cervical lesions.

The present study aimed to investigate HPV prevalence, HPV genotype and HPV-16 variants distribution among women attending Women’s Healthcare Centers in the central region of Tunisia. This study would provide background information to improve cervical cancer screening and to predict the impact of HPV vaccination program in an Arab Muslim country.

## Methods

### Study population and samples

The National Ethical Committee approves this cross-sectional study. Prior to their participation, written informed consent was obtained from all enrolled women. Participants were collected from Public (National Office of Family and Population and Department of Gynecology and Obstetrics) and Private Women’s Healthcare Centers located at Monastir and Sousse. These locations are two neighboring coastal towns known for their touristic activity that share similar lifestyle habits and sociological level. Inclusion criteria include sexually active (≥1 year) and non-pregnant women. Exclusion criteria included immunocompromised patients, patients under corticosteroid therapy, hysterectomized women and previous surgical procedures on the cervix. The cervical sampling was performed according to routine clinical methods and standards. Trained gynecologist or midwife collected samples using sterile speculum. A first sample for cytological screening was collected by the Ayer spatula. Cytological smears were reported by cytopathologists according to the 2001 Bethesda system. A second sample for molecular HPV screening was collected by a cytobrush and placed in 5 mL of phosphate buffered saline (PBS).

All subjects were interviewed, during gynecologic visit, using a questionnaire to collect demographic characteristics, medical history, contraceptive methods, sexual and reproductive behavior information and women’s lifestyle (age at first intercourse, number of sexual partners, smoking, alcohol consumption) and knowledge about HPV screening and vaccine.

### HPV-DNA detection

Cervical cells were recovered from the cervical cytobrush specimen by centrifugation and viral DNA was extracted using QIAamp MinElute virus spin kit (Qiagen, CA, USA) according to the manufacturer’s instructions. Concentration and purity of the extracted DNA were evaluated using a NanoDrop spectrophotometer, according to the manufacture’s instructions (Thermo Fisher Scientific—Nano Drop, USA). To detect HPV-DNA, real-time polymerase chain reaction (RT-PCR) assay was performed using MY09/MY11 primers and SYBR Green I chemistry [23]. Theses primers allow amplification of highly conserved 450 bp sequence of the *L1* gene. To ensure the DNA quality of the samples, a second primer pair of PCO3/PCO4 that provides amplification of 100 bp sequence of the *β-globin* gene was used simultaneously as an internal control for each PCR reaction. One hundred nanograms of the extracted cervical DNA was added to a mixture containing 12.5 μL of iQ™ SYBR®Green Supermix (BioRad, France), 0.64 μM of each primer MY09/MY11, 0.08 μM of each primer PCO3/PCO4 and nuclease free water yielding a final volume of 25 μL. The reaction was carried out on an iQ5 thermocycler (BioRad, France) using the following conditions: initial denaturation at 94 °C for 5 min followed by 30 cycles of denaturation at 94 °C for 30 s, annealing primers at 55 °C for 1 min and primers extension at 72 °C for 90s. A positive control (cloned HPV-DNA) and a negative control (nuclease free water) were included in each amplification reaction. HPV positive samples were detected using dissociation curve, which gave melting peaks at 81 ± 0.5 °C. For confirmation, PCR products were run on a 2% agarose gel stained with ethidium bromide and visualized with UV light.

### HPV genotyping and variants analysis

HPV-DNA positive samples were subjected to DNA sequencing to identify HPV types and HPV-16 variants. The gel purified L1-PCR products were sequenced using BigDyeDeoxy terminator cycle sequencing kit (BD V3.1, Applied Biosystems) according to the manufacturer’s instructions. The products were then purified on a separation column (AutoSeq™ G-50, Amersham Biosciences), and the templates were sequenced on an automated ABI-PRISM 310 Genetic Analyzer (Applied Biosystems). Sequence analysis was performed using nucleotide-nucleotide BLAST analysis (blastn) against known HPV genotype and HPV-16 reference variants sequences stored in the GenBank database (www.ncbi.nlm.nih.gov/BLAST, June 2015) (Human papillomavirus type 16, complete genome, GenBank: K02718.1; Human papillomavirus type 16 variant (African type 1), complete genome, GenBank: AF472508.1; Human papillomavirus type 16 variant (African type 2), complete genome, GenBank: AF472509.1; Human papillomavirus type 16 Asian-American variant, complete genome, GenBank: AF402678.1).

### Statistical analysis

Statistical analysis was performed using the SPSS Statistical package (IBM SPSS Statistics version 21.0). The distribution of age, age at first intercourse and years of sexual activity, according to the different study population subgroups were analyzed and a t-test was used to compare these quantitative variables. The Chi-square test was used to estimate the differences in HPV prevalence between the groups of studied women. The Spearman’s Rank correlation test was used to test the relationship between age and age at first intercourse.

A descriptive analysis and frequency distributions were conducted for all studied risk factors associated with HPV infection and squamous intraepithelial lesion (SIL) occurrence (age, age at first intercourse, years of sexual activity, marital status, number of sexual partners, number of pregnancies, contraceptive method’s use, menopausal status, Smoking and/or alcohol consumption and education level) in order to calculate sample size and percentages. Possible associations between HPV positivity or SIL and all the above-cited factors were determined by logistic regression to estimate Odds ratios (ORs) and 95%-confidence intervals (95%-CIs). Variables with a *p*-value less than 0.05 in the univariate analysis were included in the multivariate analysis. A *p-*value of less than 0.05 was considered significant.

## Results

### Study population characteristics

In this study, no significant difference was observed when demographic data and distribution of lifestyle factors of the included women from the different locations were compared (Monastir and Sousse). Among the 494 eligible women, 23 DNA samples were negative for β-globin amplification and were consequently excluded from further analysis. Data were thus evaluated for 471 cases. Demographic, lifestyle and clinical characteristics of these women were summarized in Table 1. Women are aged from 17 to 73 years (median, 35 years) and 66.1% are in the age group of 26 to 45 years. The median age at first intercourse was 22 years (range, 12 to 48 years) and the median period of sexual activity was 12 years (range, 1 to 47 years). To show if there is a change in sexual behavior over the different generations, we evaluated using the Spearman’s Rank correlation test the correlation between age and age at first intercourse. We found that among younger women (≤40 years), age at first intercourse was positively correlated with their age (*R* = 0.356; *p* < 10^-6^). While, among more aged women (>40 years) a negative correlation was observed (*R* = -0.068; *p* > 0.05). The majority of women have primary or secondary education (80%); 10% have received higher education; and 10% were illiterate. The participating women are mainly non menopausal (90.9%); 16.8% of them use hormonal contraception and only 1% use condom as a contraceptive method. Most of the women are married (76.2%) and 22.3% declared that they have more than one sexual partner. Vaginal bacterial infections were observed among 10% (vaginal candidiasis and trichomoniasis). Based on women’s medical files, data about viral sexually transmitted infections (HSV, HCV and HIV) were available only for 152 participants and no positive results were reported. None of the enrolled women have ever been screened for HPV infection or received HPV vaccine or heard about the HPV vaccine. Pap smear cytology results show that 15.9% (*n* = 75) of women have HPV associated lesions (SIL) and 84.1% (*n* = 396) of them have no intraepithelial lesion (NIL). Women with SIL are classified into 41.3% with low-grade SIL (LSIL) (*n* = 31) and 58.7% with high-grade SIL (HSIL) (*n* = 44).

**Table 1:** Epidemiologic and lifestyle characteristics of the study population

Characteristic	Total *n* = 471 (%)
Age (years)	
≤ 25	53 (11.2)
26–35	183 (38.9)
36–45	128 (27.2)
46–55	79 (16.8)
≥ 56	28 (5.9)
Age at first intercourse (years)	
< 20	123 (26.1)
20–24	213 (45.2)
≥ 25	135 (28.7)
Years of sexual activity	
≤ 10	200 (42.5)
11–20	149 (31.6)
≥ 21	122 (25.9)
Marital status	
Married	359 (76.2)
Unmarried	112 (23.8)
Number of pregnancies	
0	21 (4.4)
1–3	256 (54.4)
≥ 4	194 (41.1)
Contraceptive use	
No	318 (67.5)
Hormonal	79 (16.8)
Non hormonal	74 (15.7)
Menopausal status	
Non menopausal	424 (90.9)
Menopausal	47 (9.1)
Number of sexual partner	
1	366 (77.7)
≥ 2	105 (22.3)
Smoking/alcohol consumption	
No	386 (82)
Yes	85 (18)
Education level	
Illiterate	49 (10.4)
Literate	422 (89.6)

### HPV prevalence

The overall HPV prevalence assessed by real-time PCR amplification and melting curve analysis was 34% (*n* = 160, 95% CI: 30–38%) with significantly higher prevalence among women with SIL than those with NIL 84% (*n* = 63, 95% CI: 76–92%) and 24.5% (*n* = 97, 95% CI: 20–29%) respectively, *p* < 10^-6^) (Table 2). Furthermore, the HR-HPV prevalence was four times higher among women with SIL than women with NIL. HPV prevalence was not significant between women with LSIL and HSIL (87.1%, 95% CI: 75–100% and 81.8%, 95% CI: 70–94%, respectively). However, HR-HPV prevalence rate was about two times higher among women with HSIL compared to women with LSIL (70%, 95% CI: 55–85%, and 42.3%, 95% CI: 22–63%, respectively, *p* = 0. 025). Moreover, as expected, NIL women having multiple sexual partners had a significantly higher HPV prevalence than NIL women having one sexual partner (42.4% (*n* = 42, 95% CI: 33–52%) and 18.5% (*n* = 55, 95% CI: 14–23%) respectively, *p* = 3.10^-5^). The HR-HPV prevalence rate was about three times higher among women who have multiple sexual partners than those who have one sexual partner (31.5%, 95% CI: 22–41% and 9.4%, 95% CI: 6–13%, respectively, *p* < 10^-6^).

**Table 2:** HPV prevalence among women with normal cytology and squamous intraepithelial lesion

	No Intraepithelial Lesion	Squamous Intraepithelial Lesion				
	Total (*n* = 396)	Total (*n* = 75)	LSIL (*n* = 31)	HSIL (*n* = 44)	*p* OR [95% CI]	*p’* OR [95% CI]
HPV positive women	97	63	27	36		
HPV prevalence (%)	24.5	84	87.1	81.8	0.541	<10^-6^
					1.5 [0.409–5.503]	16.183 [8.377–31.262]
HR-HPV positive women	55	39	11	28		
HR-HPV prevalence (%)	14.9	59.1	42.3	70	0.025	<10^-6^
					3.181[1.135–8.920]	8.273 [4.687–14.602]

*LSIL*, low-grade squamous intraepithelial lesion; *HSIL*, high-grade squamous intraepithelial lesion; *p,* results of LSIL *vs* HSIL comparison; *p’,* results of NIL *vs* SIL comparison

**Fig. 1:**
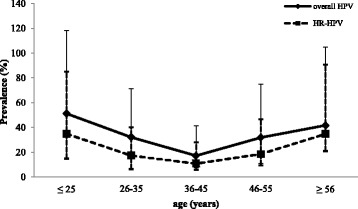
Distribution of age specific HPV and HR-HPV prevalence and their corresponding 95% confidence interval (CI)

We then analyzed the prevalence of HPV infection according to the age groups of women (Fig. 1). The distribution of HPV prevalence, showed a U-shaped curve with peaks of HPV prevalence among the youngest women (≤25 years; 51.2%, 95% CI: 37–67%) and the oldest women (>55 years; 41.7%, 95% CI: 20–63%). The lowest HPV prevalence was observed among women aged from 36 to 45 years (17.2%, 95% CI: 10–24%). Moreover, the distribution of HR-HPV prevalence according to age groups shows the same pattern as overall HPV prevalence (Fig. 1). Highest HR-HPV prevalence rates were observed among the youngest aged women (≤25 years; 34.9%, 95% CI: 20–50%), and among the oldest aged women (>55 years; 34.8%, 95% CI: 14–56%).

### HPV genotype and HPV-16 variants distribution

HPV-DNA positive samples were analyzed by direct sequencing to identify HPV genotype distribution in the study population. Out of the 160 samples positive for HPV infection, 35 samples were lacking in HPV genotyping because of the poor quality of the cervical DNA. As shown in Table 3, 18 different HPV types were identified in our population and five cases of co-infections were also observed. Among the 125 HPV-positive samples analyzed, HPV-16 was the most frequent genotype, accounting for 44.8%, (*n* = 56, 95% CI: 20–29%) followed by HPV-6 (11.2%, 95% CI: 6–17%), HPV-11 (7.2%, 95% CI: 3–12%), HPV-31 (5.6%, 95% CI: 2–10%), HPV-58 (5.6%, 95% CI: 2–10%) and HPV-66 (4.8%, 95% CI: 1–9%). However, HPV-18 prevalence rate was less than 2% (95% CI: 0–4%). Among the nine HR-HPV types detected, HPV-45 was the least frequent type, which was detected in only one case of HSIL. HR HPV types were more prevalent among HPV positive women having multiple sexual partners and HSIL (23.2%, 95% CI: 16–31%, and 22.4%, 95% CI: 15–30%, respectively). HPV-16 was the most prevalent HR type among women with NIL and SIL (9.2%, 95% CI: 6–12% and 32.8%, 95% CI: 22–45%, respectively), it was also frequent among women having multiple sexual partners and HSIL (12.1%, 95% CI: 5–19% and 43.9%, 95% CI: 29–61%, respectively). Concerning LR-HPV types, they were more frequent among LSIL women (42.3%, 95% CI: 22–63%) (Table 3).

**Table 3:** HPV genotypes distribution among women with normal cytology and squamous intraepithelial lesion

HPV type	Whole HPV	No Intraepithelial Lesion	Squamous Intraepithelial Lesion		
		Total	Total	LSIL	HSIL
HR-HPV	85	48	37	11	26
HPV-16	56	34	22	4	18
HPV-18	2	1	1	0	1
HPV-31	7	3	4	3	1
HPV-33	3	1	2	0	2
HPV-45	1	0	1	0	1
HPV-56	4	0	4	3	1
HPV-58	7	4	3	1	2
HPV-68	3	3	0	0	0
HPV-82	2	2	0	0	0
Probably HR-HPV	9	7	2	0	2
HPV-53	3	3	0	0	0
HPV-66	6	4	2	0	2
LR-HPV	31	16	15	11	4
HPV-6	14	5	9	5	4
HPV-11	9	3	6	6	0
HPV-61	1	1	0	0	0
HPV-70	1	1	0	0	0
HPV-81	1	1	0	0	0
HPV-83	1	1	0	0	0
HPV-84	4	4	0	0	0
Co-infection	5	2	3	2	1
HPV-6/11	2	1	1	1	0
HPV-11/18	1	1	0	0	0
HPV-11/31	1	0	1	1	0
HPV-16/18	1	0	1	0	1

*LSIL*, low-grade squamous intraepithelial lesion; *HSIL*, high-grade squamous intraepithelial lesion

In the current study, molecular characterization of HPV-16 variants was performed using MY09/MY11-L1 region. Three different HPV-16 variants, European (E), African-2 (Af-2) and Asian-American (AA), were detected in our population study. None of the HPV-16 positive cases belongs to African-1 variant. HPV-16 E variant was the most frequent (85.7%, 95% CI: 76–95%). The prevalence of the HPV-16 E variant was highest in HSIL (34.2%, 95% CI: 20–50%), followed by LSIL (15.4%, 95% CI: 1–30%), NIL (8.13%, 95% CI: 5–11%). The majority of HPV-16 Af-2 variant was found in HSIL than NIL (9.8%, 95% CI: 0–20%), 0.5%, 95% CI: 0–1%, respectively). The HPV-16 AA variant was detected only among women with NIL (0.5%, 95% CI: 0–1%).

### Risk Factors for HPV infection

In univariate analysis, the association between HPV infection and several risk factors among our study population were assessed (Table 4). The results showed that, HPV and HR-HPV infection was significantly associated with women’s lifestyle (marital status, number of sexual partners and smoking/alcohol consumption) and young age. However, other risk factors such as age at first intercourse, number of pregnancies, contraceptive methods, menopausal status and education level were not associated with HPV or HR-HPV infection. The risk factors with a *p*-value less than 0.05 from univariate analysis were further included in multivariate analysis (Table 4). These results showed that, only young (<30 years) age was an independent factor associated with HPV infection. Moreover, it significantly increases the risk of HR-HPV infection. Number of sexual partners was not found to be an independent risk factor for HPV and HR-HPV infections in our study population.

**Table 4:** Univariate and multivariate logistic regression analysis of risk factors for HPV infection

Risk Factors	Risks for HPV infection (*n* = 471)			Risks for HR-HPV infection (*n* = 436)^a^		
	Univariate analysis		Multivariate analysis	Univariate analysis		Multivariate analysis
	HPV prevalence (%)	*p*	OR [95% CI]	HR-HPV prevalence (%)	*p’*	OR [95% CI]
Age						
≤ 30	41.1		1	24.8		1
> 30	26.4	0.002	0.51 [0.33–0.79]	16	0.041	0.57 [0.34–0.98]
Marital status						
Married	28.8			16.6		
Unmarried	41.7	0.012	ns	31.7	0.001	ns
Number of sexual partners						
1	28.5			16.5		
≥ 2	43.6	0.004	ns	33	4.10^-4^	ns
Smoking/alcohol consumption						
No	23.7			14.5		
Yes	46.8	4.10^-5^	ns	36	1.10^-5^	ns

*ns, * not significant; *p*, probability for HPV infection; *p’*, probability for HR-HPV infection

^a^study population without samples lacking in HPV genotyping (*n* = 35)

### Risk Factors for SIL occurrence

When we compared epidemiological, demographic, and lifestyle habits of women with NIL and SIL, we found that women with SIL had a higher HPV prevalence, a younger age at first intercourse and a longer period of sexual activity (*p* < 10^-6^, *p* = 0.008 and *p* = 0.010, respectively). In addition, when we compared characteristics of LSIL women with HSIL women, we found that those with HSIL had a significantly higher HR-HPV prevalence, were older and had a longer period of sexual activity (*p* = 0.025, *p* = 0.004 and *p* = 0.010, respectively). No more significant differences were found between women with NIL and SIL or between HSIL and LSIL.

Furthermore, we conducted univariate and multivariate analysis to determine the association between risk of SIL or HSIL occurrence and different putative risk factors (Table 5). The univariate analysis results show that, as expected, HPV infection was the main significant associated risk factor, together with menopause for SIL. However, no statistically significant associations were observed with women’s age, age at first intercourse, period of sexual activity, the number of pregnancies, contraceptive methods, smoking/alcohol consumption and education level. In univariate analysis, we found significant association between HSIL and HPV infection, period of sexual activity longer than 10 years and menopause. While, in multivariate analysis, HPV infection, and menopause were independent risk factors for both SIL and HSIL (Table 5).

**Table 5:** Univariate and multivariate logistic regression analysis of risk factors for squamous intraepithelial lesion and high-grade squamous intraepithelial lesion

Risk Factors	Risks for SIL (*n* = 471)			Risks for HSIL (*n* = 471)		
	Univariate analysis		Multivariate analysis	Univariate analysis		Multivariate analysis
	SIL (%)	*P*	OR [95% CI]	HSIL (%)	*P’*	OR [95% CI]
HPV status						
HPV negative	3.9		1	2.6		1
HPV positive	39.4	<10^-6^	14.10 [7.00–28.40]	22.5	<10^-6^	11.06 [4.61–26.56]
Years of sexual activity						
≤ 10	10.3			4.8		
> 10	16.5	0.080	ni	11.6	0.020	ns
Menopausal status						
Non menopausal	12		1	6		1
Menopausal	34.1	6.10^-5^	3.66 [1.62–8.29]	31.8	<10^-6^	7.30 [3.11–17.13]

*n, * number; *ns,* not significant; *ni,* not included in the multivariate analysis; *SIL*, squamous intraepithelial lesion; *HSIL*, high-grade squamous intraepithelial lesion; *p*, probability for SIL; *p’*, probability for HSIL

## Discussion

Since the commercialization of preventive HPV vaccines introduced in 2006, limited studies have been performed on molecular epidemiology of HPV infection among Tunisian women. The first molecular studies published results on the HPV infection among women with normal cervical cytology having different lifestyle practices [20, 21]. Recently, HPV type and variants distribution have been assessed in cervical tumor tissues from Tunisian patients [24]. However, there are insufficient data on the risk factors for HPV infection and cervical intraepithelial lesions. Tunisia is the only country in the Arab Muslim ones where polygamy is forbidden by law since 1956 and where the minimum legal age of marriage for females is the highest (18 years) in the Extended Middle East and North Africa (EMENA) region. Moreover, the male circumcision is a generalized practice among Muslim countries including Tunisia [25]. All these reasons could contribute to the reduced risk of HPV associated lesions among Tunisian women. Furthermore, Tunisia had a peculiar relationship with Mediterranean Europe where populations are less conservative (colonization and immigration). Thus, Tunisia is a North-African country with distinctive characteristics from other North-African, Middle Eastern and Sub-Saharian countries, where epidemiological studies to identify risk factors should be investigated to help the establishment of a routinely HPV testing and a vaccination program.

In this study, 15.9% of the enrolled women have squamous intraepithelial lesions and the HPV prevalence among them was 84%. This rate is comparable to those reported worldwide [26]. Few studies were conducted on HPV testing in the EMENA region, and results show that HPV prevalence in precancerous cervical lesions is ranged from 15% to 100% [27]. The differences in HPV prevalence among women with cervical lesions between the studies of the EMENA region are more likely due to the use of HPV testing methods with different sensitivity (ISH, FISH, PCR, hybrid capture methods) and small sample size [27–29].

For women without cervical lesions, the HPV prevalence was 24.5%, and as expected prevalence was found to be significantly higher among those having multiple sexual partners (42.4%) compared to those having one sexual partner (18.5%) or married (19.2%). Previously, Hassen et al., published HPV prevalence in women with normal cervical cytology from the central region of Tunisia (Sousse and Monastir) [20]. They observed a higher HPV prevalence in prostitutes (39%) compared to married women (14%) [20]. Herein, the slight increase in HPV prevalence among married women could be due to the use of the q-PCR method, which is more sensitive than the conventional PCR used previously by Hassen et al., rather than the change in the lifestyle and sexual habits in both Tunisian men and women. However, because of the cultural traditions, the limitation of this study is the lack of data about the sexual partner, extramarital relationships (for wives and husbands), sexual life and practice that are still taboo subjects. Considering women with normal cervical cytology, when we compared the overall HPV prevalence among Tunisian women with that from neighboring Northern African countries, we reported that it was higher than that of Algeria (5.3%) [22], but lower than that of Morocco (34.3%) [30]. Additionally, we found relatively high HPV prevalence compared to Arab-Muslim countries from the Middle-East [31–33] but substantially lower than that reported by other studies of the Sub-Saharan countries [34, 35] and Mediterranean Europe [36, 37]. The different origins and characteristics of the studied population (age, lifestyle, and ethnicity) may be the reason of these disparities in HPV prevalence.

In our study, eighteen different HPV types were detected. Out of the eleven oncogenic types, HPV-16 was the most common in our population and six (HPV-16, -18, -31, -33, -58, and -66) were found among NIL, which was consistent with global studies in women with normal cervical cytology [38]. Among women with HSIL, the predominant genotype HPV-16 was followed by HPV-33, -58 and -66. However, in this group, HPV-18 was detected only in one case of co-infection with HPV-16. To the best of our knowledge, this is the first study, which describes HPV-16 variants distribution among Tunisian women with and without cervical intraepithelial lesions. The distribution of major variants around the world was known to be highly geographically and ethnically specific. For instance, the European variant is known to be spread worldwide, except for Sub-Saharan Africa, where the African variants are more prevalent [39]. In our cohort, HPV-16 European variant was the most common (85.7%), this may be due to the geographical location and historical links between Tunisia and the European continent. Our results showed that the rate of HPV-16 E variant was higher among NIL women than SIL women (88.2% and 81.8%, respectively) whereas, non-European variants were high among SIL women compared to NIL women (18% and 12%, respectively). KrennHrubec et al., observed that among cervical cancer cases collected from the center of Tunisia, a prevalence rate was of 61% for the HPV-16 E variant and of 40% for the non-European variants [24]. Non-European variants were also reported to be frequently observed among cervical cancer cases from Morocco [40]. Altogether, these results suggest that the prevalence of non-European variants increases with the severity of the lesion. The relationship between non-European lineage and occurrence of cervical lesions in Tunisian women should be more investigated.

We also explored risk factors influencing HPV infection and squamous intraepithelial lesions occurrence. Previous studies have shown that young age, age at the first sexual intercourse, multiple sexual partners, oral contraceptive use, smoking, number of pregnancies and menopause are the specific risk factors for cervical HPV infection [41–46]. In our study, multivariate analysis showed that only young age is independently associated with HPV infection. Moreover, numerous factors have been assessed for a putative link with SIL. This study is the first that reported risk factors for SIL occurrence for Tunisian women. Women are at increased risk of SIL and HSIL if they have HPV infection or are in menopause. The latter observation may be related to the combined effect of the persistence or the reactivation of HPV infection with weakness of the local immune response at menopause. Accordingly, Castle et al, demonstrated that HPV persistence increased significantly for post-menopausal women [47]. Hormonal disturbance of the menopause was associated with deregulated immune function; for instance the decrease of estrogen level induces a poor ability to eliminate HPV infection [48, 49]. Herein, women with SIL are older and have a younger age at first intercourse than women with NIL. Recent studies reported that both, adolescents with immature epithelium and menopausal women have an affected expression of inflammatory cytokines and chemokines in cervical samples [50, 51]. Altogether, these observations may explain the age-related HPV prevalence pattern for young and menopausal women. Thus, adding HPV testing to Pap smear screening for menopausal women may improve the detection of a persistent or a recent HPV infection.

Evaluation of prevalence and genotype distribution of HPV infection in the general population is very important to estimate the potential impact of current HPV vaccines. Currently, three vaccines against HPV infection are available and distributed in more than 100 countries (a bivalent to prevent HPV-16 and HPV-18 infection, a quadrivalent to prevent HPV-16, -18, -6 and -11 infection and a nonavalent HPV vaccine targeting HPV-16, -18, -31, -33, -45, -52, -58, -6, and -11). However, HPV type classification shows a great difference between geographic regions worldwide [52, 53]. HPV bivalent vaccine is commercially available in Tunisia, but none of the women included in this study heard about it. Our study shows that HPV types targeted by the bivalent vaccine were found among 13% of the women. Thus, in Tunisia, there is a need to establish advertisement and public campaigns for HPV vaccine and to consider the quadrivalent and nonavalent HPV vaccines as alternatives to the bivalent vaccine.

## Conclusion

This cross-sectional study contributes to the knowledge of HPV epidemiology in Tunisian women with and without cervical lesions. We found that infected menopausal women were at higher risk to develop cervical lesions. We also found that the HPV-16 was dominant and that the HPV-18 was not very common, suggesting that the currently available bivalent vaccine may have lower efficiency with Tunisian women. Altogether, these results may be helpful for designing an adapted HPV testing and vaccination program in Tunisia.

## Abbreviations

AA: Asian-American variant
Af-1: African-1 variant
Af-2: African-2 variant
CC: Cervical cancer
E: European variant
EMENA: Extended Middle Eastern and North African
HPV: Human papillomavirus
HR: High-risk
LSIL: Low-grade squamous intraepithelial lesion
IARC: International Agency for Research on Cancer
LR: Low-risk
LSIL: Low-grade squamous intraepithelial lesion
NIL: No intraepithelial lesion
PBS: Phosphate buffered saline
SIL: Squamous intraepithelial lesion

## References

[1] Ferlay J, Soerjomataram I, Ervik M, Dikshit R, Eser S, Mathers C, Rebelo M, Parkin DM, Forman D, Bray F (2013). GLOBOCAN 2012 v1.0, Cancer Incidence and Mortality Worldwide: IARC Cancer Base No. 11 [Internet].

[2] International Agency for Research on Cancer (IARC) (1995). IARC Monographs on the evaluation of carcinogenic risks of humans.

[3] Schiffman M, Castle PE, Jeronimo J, Rodriguez AC, Wacholder S (2007). Human Papillomavirus and cervical cancer. Lancet.

[4] Favre M (1975). Structural polypeptides of rabbit, bovine, and human papillomavirus. J Virol.

[5] zur Hausen H (1985). Genital papillomavirus infections. Prog Med Virol.

[6] de Villiers EM, Fauquet C, Broker TR, Bernard HU, zur Hausen H (2004). Classification of papillomaviruses. Virology.

[7] HPV Center. International Human Papillomavirus Reference Center. Davit Bzhalava, Karolinska Institutet. 2015. http://www.hpvcenter.se/html/refclones.html. Accessed 11 Feb 2015.

[8] Terai M, Burk RD (2002). Identification and characterization of 3 novel genital human papillomaviruses by overlapping polymerase chain reaction: candHPV89, candHPV90, and candHPV91. J Infect Dis.

[9] Muñoz N, Bosch FX, de Sanjosé S, Herrero R, Castellsagué X, Shah KV, Snijders PJ, Meijer CJ, International Agency for Research on Cancer Multicenter Cervical Cancer Study Group (2003). Epidemiologic classification of human Papillomavirus types associated with cervical cancer. N Engl J Med.

[10] Schiffman M, Cliffford G, Buonaguro FM (2009). Classification of weakly carcinogenic human papillomavirus types: Addressing the limits of epidemiology at the borderline. Infect Agent Cancer.

[11] Chen Z, Terai M, Fu L, Herrero R, DeSalle R, Burk RD (2005). Diversifying selection in human papillomavirus type 16 lineages based on complete genome analyses. J Virol.

[12] Sichero L, Ferreira S, Trottier H, Duarte-Franco E, Ferenczy A, Franco EL, Villa LL (2007). High grade cervical lesions are caused preferentially by non-European variants of HPVs 16 and 18. Int J Cancer.

[13] Schiffman M, Rodriguez AC, Chen Z, Wacholder S, Herrero R, Hildesheim A (2010). A population-based prospective study of carcinogenic human papillomavirus variant lineages, viral persistence, and cervical neoplasia. Cancer Res.

[14] Herrero R, Hildesheim A, Bratti C, Sherman ME, Hutchinson M, Morales J (2000). Population-based study of human papillomavirus infection and cervical neoplasia in rural Costa Rica. J Natl Cancer Inst.

[15] Lazcano-Ponce E, Herrero R, Muñoz N, Cruz A, Shah KV, Alonso P, Hernández P, Salmerón J, Hernández M (2001). Epidemiology of HPV infection among Mexican women with normal cervical cytology. Int J Cancer.

[16] Baseman JG, Koutsky LA (2005). The epidemiology of human Papillomavirus infections. J ClinVirol.

[17] de Sanjosé S, Diaz M, Castellsagué X, Clifford G, Bruni L, Muñoz N (2007). Worldwide prevalence and genotype distribution of cervical human papillomavirus DNA in women with normal cytology: a meta-analysis. Lancet Infect Dis.

[18] Almonte M, Albero G, Molano M, Carcamo C, García PJ, Pérez G (2008). Risk factors for human papillomavirus exposure and co-factors for cervical cancer in Latin America and the Caribbean. Vaccine.

[19] Vaccarella S, Franceschi S, Herrero R, Muñoz N, Snijders PJ, Clifford GM (2006). Sexual behavior, condom use, and human papillomavirus: pooled analysis of the IARC human papillomavirus prevalence surveys. Cancer Epidemiol Biomarkers Prev.

[20] Hassen E, Chaieb A, Letaief M, Khairi H, Zakhama A, Remadi S (2003). Cervical human papillomavirus infection in Tunisian women. Infection.

[21] De Marco F, Houissa-Kchouk F, Khelifa R, Marcante ML (2006). High-risk HPV types in Tunisia. A pilot study reveals an unexpectedly high prevalence of types 58 and 82 and lack of HPV 18 among female prostitutes. J Med Virol.

[22] Hammouda D, Clifford GM, Pallardy S, Ayyach G, Chékiri A, Boudrich A (2011). Human papillomavirus infection in a population-based sample of women in Algiers, Algeria. Int J Cancer.

[23] Manos MM, Ting Y, Wright DK, Lewis AJ, Broker TR, Wolinsky SM (1989). The use of polymerase chain reaction amplification for the detection of the human papillomaviruses. Cancer Cells.

[24] KrennHrubec K, Mrad K, Sriha B, Ben Ayed F, Bottalico DM, Ostolaza J (2011). HPV types and variants among cervical cancer tumors in three regions of Tunisia. J Med Virol.

[25] Castellsagué X, Bosch FX, Muñoz N, Meijer CJ, Shah KV, de Sanjose S (2002). Male circumcision, penile human papillomavirus infection, and cervical cancer in female partners. N Engl J Med.

[26] Forman D, de Martel C, Lacey CJ, Soerjomataram I, Lortet-Tieulent J, Bruni L (2012). Global burden of human papillomavirus and related diseases. Vaccine.

[27] Seoud M (2012). Burden of human papillomavirus-related cervical disease in the extended middle East and north Africa-a comprehensive literature review. J Low Genit Tract Dis.

[28] Kleter B, van Doorn LJ, Schrauwen L, Molijn A, Sastrowijoto S, ter Schegget J (1999). Development and clinical evaluation of a highly sensitive PCR-reverse hybridization line probe assay for detection and identification of anogenital human papillomavirus. J Clin Microbiol.

[29] Snijders PJ, van den Brule AJ, Meijer CJ (2003). The clinical relevance of human papillomavirus testing: relationship between analytical and clinical sensitivity. J Pathol.

[30] Souho T, El Fatemi H, Karim S, El Rhazi K, Bouchikhi C, Banani A (2016). Distribution of carcinogenic human papillomavirus genotypes and association to cervical lesions among women in Fez (Morocco). PLoS One.

[31] Al-Ahdal MN, Al-Arnous WK, Bohol MF, Abuzaid SM, Shoukri MM, Elrady KS (2014). Human papillomavirus in cervical specimens of women residing in Riyadh, Saudi Arabia: a hospital-based study. J Infect Dev Ctries.

[32] Bansal D, Elmi AA, Skariah S, Haddad P, Abu-Raddad LJ, Al Hamadi AH (2014). Molecular epidemiology and genotype distribution of Human Papillomavirus (HPV) among Arab women in the State of Qatar. J Transl Med.

[33] Al-Awadhi R, Chehadeh W, Kapila K (2011). Prevalence of human Papillomavirus among women with normal cervical cytology in Kuwait. J Med Virol.

[34] Manga MM, Fowotade A, Abdullahi YM, El-Nafaty AU, Adamu DB, Pindiga HU (2015). Epidemiological patterns of cervical human papillomavirus infection among women presenting for cervical cancer screening in North-Eastern Nigeria. Infect Agent Cancer.

[35] Zoa Assoumou S, Ndjoyi Mbiguino A, Mabika Mabika B, Nguizi Ogoula S, El Mzibri M, Khattabi A (2016). Human papillomavirus genotypes distribution among Gabonese women with normal cytology and cervical abnormalities. Infect Agent Cancer.

[36] Casalegno JS, Benchaib M, Le Bail Carval K, Piaton E, Mathevet P, Mekki Y (2011). Human papillomavirus genotype distribution among French women with and without cervical abnormalities. Int J Gynaecol Obstet.

[37] Piana A, Sotgiu G, Castiglia P, Pischedda S, Cocuzza C, Capobianco G (2011). Prevalence and type distribution of human papillomavirus infection in women from North Sardinia, Italy. BMC Public Health.

[38] Bruni L, Diaz M, Castellsagué X, Ferrer E, Bosch FX, de Sanjosé S (2010). Cervical human papillomavirus prevalence in 5 continents: meta-analysis of 1 million women with normal cytological findings. J Infect Dis.

[39] Cornet I, Gheit T, Iannacone MR, Vignat J, Sylla BS, Del Mistro A, Franceschi S, Tommasino M, Clifford GM (2013). HPV16 genetic variation and the development of cervical cancer worldwide. Br J Cancer.

[40] Qmichou Z, Khyatti M, Berraho M, Ennaji MM, Benbacer L, Nejjari C (2013). Analysis of mutations in the E6 oncogene of human papillomavirus 16 in cervical cancer isolates from Moroccan women. BMC Infect Dis.

[41] Burk RD, Ho GY, Beardsley L, Lempa M, Peters M, Bierman R (1996). Sexual behavior and partner characteristics are the predominant risk factors for genital human papillomavirus infection in young women. J Infect Dis.

[42] Flores YN, Bishai DM, Shah KV, Lazcano-Ponce E, Lörincz A, Hernández M (2008). Risk factors for cervical cancer among HPV positive women in Mexico. Salud Publica Mex.

[43] Zhang R, Shi TY, Ren Y, Lu H, Wei ZH, Hou WJ (2013). Risk factors for human papillomavirus infection in Shanghai suburbs: a population-based study with 10,000 women. J Clin Virol.

[44] Jahdi F, Khademi K, Khoei EM, Haghani H, Yarandi F (2013). Reproductive factors associated to human papillomavirus infection in Iranian woman. J Family Reprod Health.

[45] Ribeiro AA, Costa MC, Alves RR, Villa LL, Saddi VA, Carneiro MA (2015). HPV infection and cervical neoplasia: associated risk factors. Infect Agent Cancer.

[46] Marks MA, Gupta S, Liaw KL, Tadesse A, Kim E, Phongnarisorn C (2015). Prevalence and correlates of HPV among women attending family-planning clinics in Thailand. BMC Infect Dis.

[47] Castle PE, Schiffman M, Herrero R, Hildesheim A, Rodriguez AC, Bratti MC (2005). A prospective study of age trends in cervical human papillomavirus acquisition and persistence in Guanacaste, Costa Rica. J Infect Dis.

[48] Straub RH (2007). The complex role of estrogens in inflammation. Endocr Rev.

[49] Hale GE, Burger HG (2009). Hormonal changes and biomarkers in late reproductive age, menopausal transition and menopause. Best Pract Res Clin Obstet Gynaecol.

[50] Hwang LY, Scott ME, Ma Y, Moscicki AB (2011). Higher levels of cervicovaginal inflammatory and regulatory cytokines and chemokines in healthy young women with immature cervical epithelium. J Reprod Immunol.

[51] Sivro A, Lajoie J, Kimani J, Jaoko W, Plummer FA, Fowke K (2013). Age and menopause affect the expression of specific cytokines/chemokines in plasma and cervical lavage samples from female sex workers in Nairobi, Kenya. Immun Ageing.

[52] Clifford GM, Smith JS, Aguado T, Franceschi S (2003). Comparison of HPV type distribution in high-grade cervical lesions and cervical cancer: a meta-analysis. Br J Cancer.

[53] Orozco-Colín A, Carrillo-García A, Méndez-Tenorio A, Ponce-de-León S, Mohar A, Maldonado-Rodríguez R (2010). Geographical variation in human papillomavirus prevalence in Mexican women with normal cytology. Int J Infect Dis.

